# Compound Kushen Injection suppresses human breast cancer stem-like cells by down-regulating the canonical Wnt/β-catenin pathway

**DOI:** 10.1186/1756-9966-30-103

**Published:** 2011-10-28

**Authors:** Weiru Xu, Hongsheng Lin, Ying Zhang, Xinyi Chen, Baojin Hua, Wei Hou, Xin Qi, Yingxia Pei, Xiaoyun Zhu, Zhizheng Zhao, Liangliang Yang

**Affiliations:** 1 Oncology Department, Guang An Men Hospital, China Academy of Chinese Medical Sciences, No.5 Bei Xian Ge Street, Xicheng District, Beijing 100053, China; 2 Department of Hematology and Oncology, Dong Zhi Men Hospital Affiliated to Beijing University of Chinese Medicine, No. 5, Haiyuncang, Dongcheng District, Beijing 100700, China; 3 Endocrinology Department, Guang An Men Hospital, China Academy of Chinese Medical Sciences, No.5 Bei Xian Ge Street, Xicheng District, Beijing 100053, China

**Keywords:** cancer stem-like cells, side population, Compound Kushen Injection, MCF-7, Wnt/β-catenin signaling, cisplatin

## Abstract

**Background:**

Cancer stem cells (CSCs) play an important role in cancer initiation, relapse and metastasis. To date, no specific medicine has been found to target CSCs as they are resistant to most conventional therapies and proliferate indefinitely. Compound Kushen Injection (CKI) has been widely used for cancer patients with remarkable therapeutic effects in Chinese clinical settings for many years. This study focused on whether CKI could inhibit MCF-7 SP cells in vitro and in vivo.

**Methods:**

The analysis of CKI on SP population and the main genes of Wnt signaling pathway were studied first. Then we studied the tumorigenicity of SP cells and the effects of CKI on SP cells in vivo. The mice inoculated with 10,000 SP cells were randomly divided into three groups (6 in each group) and treated with CKI, cisplatin and saline (as a control) respectively for 7 weeks. The tumor formation rates of each group were compared. The main genes and proteins of the Wnt signaling pathway were analyzed by RT-PCR and western blot.

**Results:**

CKI suppressed the size of SP population (approximately 90%), and down-regulated the main genes of Wnt signaling pathway. We also determined that MCF-7 SP cells were more tumorigenic than non-SP and unsorted cells. The Wnt signaling pathway was up-regulated in tumors derived from SP cells compared with that in tumors from non-SP cells. The tumor formation rate of the CKI Group was 33% (2/6, *P *< 0.05), and that of Cisplatin Group was 50%(3/6, *P *< 0.05), whereas that of the Control Group was 100% (6/6).The RT-PCR and western blot results indicated that CKI suppressed tumor growth by down-regulating the Wnt/β-catenin pathway, while cisplatin activated the Wnt/β-catenin pathway and might spare SP cells.

**Conclusions:**

It suggested that CKI may serve as a novel drug targeting cancer stem-like cells, though further studies are recommended.

## Background

Accumulating evidence has indicted that cancer stem cells (CSCs) are the roots of oncogenesis, cancer relapse and metastasis as they are resistant to all conventional therapies, even the advanced targeted therapy [1-6]. To date, CSCs have been identified in leukemia [7], breast cancer [8], brain cancer [9], prostate cancer [10], gastrointestinal cancer [11], and other cancers with various techniques. One of them, the side population cell sorting analysis, is now capable of isolating cells which contain CSCs [12-17]. CSCs have the ability to exclude the DNA binding dye, Hoechst33342 through an adenosine triphosphate-binding cassette (ABC) membrane transporter. Recently, SP cells have been identified in multiple solid tumors and cancer cell lines including breast cancer cell line MCF-7 [12-17]. SP cells exhibit characteristics similar to CSCs because of their ability to proliferate indefinitely and to enrich more tumorigenic cells than other populations. These rare cells have the potential to survive conventional therapeutics and regenerate cancer populations, leading to relapse and metastasis. Hence, SP cells are known as cancer stem-like cells and are a target for improved cancer therapy.

Compound Kushen Injection (CKI), commonly known as the Yanshu Injection, is extracted from two herbs Kushen (*Radix Sophorae *Flavescentis) and Baituling (*Rhizoma smilacis *Glabrae) with the primary components being oxymatrine and matrine [18]. The fingerprint of CKI is provided as additional file 1. CKI has been extensively used alone for cancer patients or in combination with chemotherapy or radiotherapy in Chinese clinical settings for many years. Previous clinical studies have shown that CKI attenuates side effects of chemotherapy and radiotherapy by improving the quality of life, regulating the immune function of cancer patients and synergizes the therapeutic effects of chemotherapy and radiotherapy as well [19,20]. It has been demonstrated that CKI suppresses tumor cell growth by inducing apoptosis [21] and inhibits the migration, invasion and adhesion capacity by down-regulating the expression of CD44_v6 _protein [22]. However, the underlying anti-cancer mechanisms are not fully understood.

The abnormal activation of the Wnt/β-catenin signaling pathway and subsequent upregulation of β-catenin driven downstream targets -- c-Myc and CyclinD1 is associated with the development of breast cancer [23]. Recent studies indicate that the Wnt/β-catenin signaling pathway also plays an important role in the maintenance of CSCs [24-27]. In addition, Wnt signaling pathway is also activated in SP breast cancer cells in vitro [14,27]. Accordingly, in order to know the importance of Wnt signaling pathway in the tumorigenicity of SP cells, the key regulators of the Wnt signaling pathway from tumors derived from both SP and non-SP cells were tested.

Our initial study revealed that the main component of CKI, oxymatrine, can decrease both MCF-7 cell viability and the size of the SP (by approximately 90%) by inhibiting β-catenin, the main component of the Wnt signaling pathway, in a dose-dependent manner, while cisplatin (DDP) only inhibits non-SP cells and spares SP cells in vitro [28]. However, studies of CKI therapy on the regulation of SP cells have never been evaluated. So we studied the effects of CKI on the treatment of SP cells and its mechanism.

## Methods

### Cell culture

Breast cancer cell line MCF-7 was kindly donated by Prof. Shuren Zhang (Department of Immunology, Cancer Institute, Peking Union Medical College and Chinese Academy of Medical Sciences). MCF-7 cells were maintained in RPMI1640 culture (Invitrogen) supplemented with 10% fetal bovine serum (Hyclone), 100 units/ml penicillin G, and 100 μg/ml streptomycin. All cells were cultured at 37°C in a humidified atmosphere containing 5% CO_2_.

### SP cell isolation

Cells were detached from cell culture flasks with 0.25% trypsin, and viable cells were counted with trypan blue and collected for inoculation into NOD/SCID mice. The remaining cells were stained with the fluorescent dye Hoechst 33342 (Sigma) at a concentration of 5 μg/mL (37°C for 90 min) as described by Goodell et al.[29] After washing with HBSS/2% FBS, the cells were incubated with 1 μg/ml propidium iodide to exclude dead cells, cell analysis and sorting were performed on a FACS Vantage SE (Becton Dickinson) by using a dual-wavelength analysis (blue, 420-470 nm; red, 660- 680 nm). We collected both MCF-7 SP and non-SP cells for the experiment.

### Tumor formation in an animal model and drug intervention

For the tumor formation assay, the NOD/SCID female mice (5-6 weeks old) were purchased from the Animal Institute of Peking Union Medical College and maintained under standard conditions according to the guidelines of the Institutional Animal Care and Use Committee of Peking University. The mice were allowed to adapt to the new environment for one week. We first identified the tumorigenicity of SP cells. Unsorted, SP and non-SP cells were collected, and cells were resuspended in PBS/Matrigel (BD Biosciences) (1:1) ranging from 10^3 ^to 5 × 10^6 ^cells per 100 μl. Cells were then injected s.c. into the bilateral mammary pads of the mice. The mice were received an estradiol supplement (0.4 mg/kg s.c., Sigma) every 10 days until the end of the experiment after cell injection. The mice without tumors were examined visually everyday. Throughout the study, mice were weighed and tumors were measured with a caliper twice a week. Tumor volumes were calculated using the formula (length×width^2^/2). When the xenograft tumors grew to proper size, the mice were euthanized and a portion of the s.c. tumor tissue was collected, fixed in 4% formalin, and embedded in paraffin for H&E staining to assess tumor pathology.

For the drug administration assay, an identical protocol was followed. The mice were randomized into three groups (6 in each group). SP cells were resuspended in PBS/Matrigel (BD Biosciences) (1:1) with 1 × 10^4 ^cells per 100 μl. 1 × 10^4 ^cells were then injected s.c. into the right mammary fat pad of each mouse at day 0. The CKI group was injected i.p. with CKI (courtesy of the Shanxi Zhengdong Pharmaceutical Co. LTD., Z14021230, China), (2 ml/kg, diluted with saline in a final volume of 200 ul) every two days, and the control group was administered with the same volume of 200 ul saline every two days beginning from 24 hours after xenotransplantation, while the DDP group was applied with DDP (courtesy of the Yunnan Supertrack Bio- pharmaceutical Corporation, H53021740, China), (5 mg/kg, diluted with saline in a final volume of 200 ul, dose according to Hardman et al.[30]) for three times at Day1, Day 8, Day 15 post inoculation.

### Quantitative RT-PCR (QRT-PCR) analysis

To assess the expression levels of β-catenin, LEF1, TCF4, CyclinD1, c-Myc, total RNA from cells/tumors was extracted by Trizol (Invitrogen) according to the manufacturer's instructions. RNA (2 μg) was quantified by spectrophotometry (DU640, Backman, USA), and reverse transcribed into cDNA using a RevertiAid™ First Strand cDNA Synthesis Kit (Fermentas, CA) according to the manufacturer's instructions. Reactions were performed using SYBR Green I Master Mix(Applied Biosystems, CA) on a GeneAmp 7500 TaqMAN PCR (Applied Biosystems, CA). PCR conditions were: initial denaturation at 95°C for 10 min followed by 40 cycles: 95°C,25 s; 55°C, 25 s and 72°C,50 s with a final extension at 72°C for 5 min. The sequences of the primers used were as follows: β-actin forward, 5'-GAGACCTTCAACACCCCAGCC-3' and reverse,

5'-AATGTCACGCACGATTTCCC-3'; β-catenin forward, 5'-AAGGTCTGAGGAGCAGCTTC-3' and reverse, 5'-TGGACCATAACTGCAGCCTT-3'; LEF1 forward, 5'-CTACCACGACAAGGCCAGAG-3' and reverse, 5'-CAGTGAGGATGGGTAGGGTTG-3' and TCF4 forward 5'-TCCCACCACATCATACGCTACAC-3', and reverse,

5'- TCGCTTGCTCTTCTCTGGACAG-3'. CyclinD1 forward, 5'-CGATGCCAACCTCCTCAACGAC-3' and reverse, 5'-CCAGCATCCAGGTGGCGACG-3' and c-Myc forward 5'-CAGCAAACCTCCTCAGCC-3', and reverse, 5'-ATTGTTTTCCAACTCCGGGAT-3'.

The amount of each target gene in a given sample was normalized to the level of β-actin in that sample. The 2^-ΔΔ*C*T ^method was applied to analyze the relative changes in gene expression [31].

### Western blot assay

Tumors were ground and lysed with the Keygen Total Protein Extraction Kit (KGP250, Keygen Serving Science, China) on ice. Tissue debris was removed by centrifugation at 4°C for 5 min. Tissue extracts were collected, and the protein concentration was determined by using the BCA Protein Assay Kit (KGPBCA, Keygen serving science, China). 60 μg of protein was run on SDS/PAGE gel and, after electrophoresis, the proteins were transferred to a PVDF membrane. Primary antibodies including anti-β-catenin (BD Bioscience, USA), anti-wnt1 (ab15251, Abcam, UK), anti-CyclinD1 (ab6125, Abcam, UK), anti-c-Myc (ab32, Abcam, UK) were applied, followed by incubation with secondary antibodies (Goat Anti-rabbit IgG, ZB2301; Goat Anti-mouse IgG, ZB2305, Zhongshan Golden Bridge Biotechnology CO., LTD., China). Blots were developed by ChemiDoc XRS System (Bio-Rad, USA).

### Statistical analysis

Student's independent-samples *t*-test, one-way ANOVA, and *χ*^2^-test were used for statistical analysis by SPSS 10.0 software (SPSS, China, 657180). *P *< 0.05 was considered significant.

## Results

### The effect of CKI on the number of SP cells in vitro

**Figure 1:**
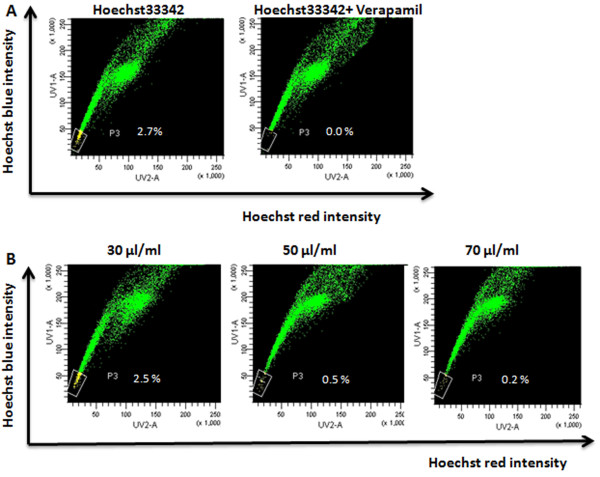
**Analysis of SP cells by CKI treatment**. (A) MCF-7 cells were labeled with Hoechst 33342 and analyzed by flow cytometry or with the addition of Verapamil. The percentage of SP cells appeared as the Hoechst low fraction in the P3 is about 2.7%. (B) MCF-7 cells were treated with CKI (30 μl/ml, 50 μl/ml, 70 μl/ml) for 48 h, and SP cells were analyzed by flow cytometry. P3 gate is the percentage of SP cells. Data from a representative experiment (from a total of three) are shown.

In Figure 1A, the P3 gate showed the SP cells with Hoechst 33342 negative/dim. SP cells accounted for approximately 2.7% of total cells. The percentage of SP population was decreased markedly by treatment with verapamil, which was consistent with the reports that verapamil could prohibit Hoechst 33342 efflux [12].

To determine whether the SP cell number decreased with CKI treatment, cells were treated with a range of concentrations of CKI (30, 50, 70 μl/ml) for 48 hours and then the SP cells were analyzed by flow cytometry. The results showed that the size of the SP population was decreased by CKI treatment in a dose-dependent manner (Figure 1B). However, our previous study didn't find the same phenomena in the cisplatin-treated cells, which were broadly used as an anti-breast cancer agent [28].

### Canonical Wnt/β-catenin pathway analysis on CKI group in vitro

**Figure 2:**
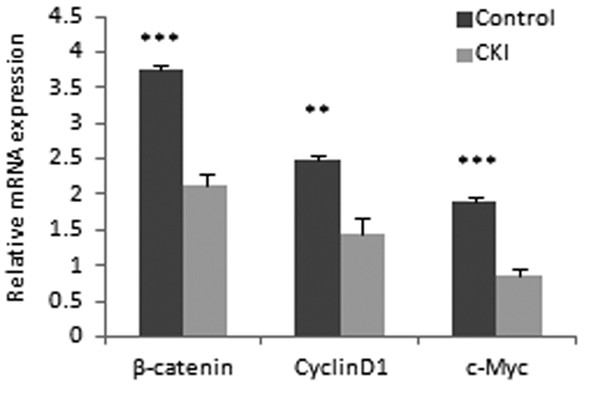
**The main genes of Wnt/β-catenin pathway was down-regulated in the CKI group in vitro**. Quantitative RT-PCR analysis revealed that the expression of β-catenin, CyclinD1 and c-Myc (mean ± SD) were lower in CKI group than those in the control group. Most of the differences were statistically significant (** *P *< 0.01,*** *P *< 0.001).

RT-PCR analysis was used to investigate whether CKI could down-regulate the expression of the main genes of Wnt/β-catenin Pathway. Sorted SP cells were treated with CKI (70 μl/ml) for 48 h and then analyzed by Quantitative RT-PCR. The study found a dramatic decrease of β-catenin, CyclinD1, c-Myc at the mRNA level with CKI treatment (Figure 2).

### SP cells are more tumorigenic in vivo

**Figure 3:**
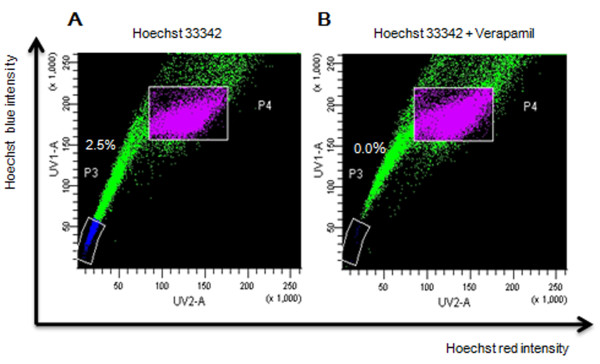
**Cell sorting results**. MCF-7 cells were labeled with Hoechst 33342 and analyzed by flow cytometry (A) or with the addition of Verapamil (B) SP cells appeared as the Hoechst low fraction in the P3 gate about 2.5%, while non-SP cells retained high levels of Hoechst staining in the P4 gate. Both SP and non-SP cells were sorted, respectively.

**Figure 4:**
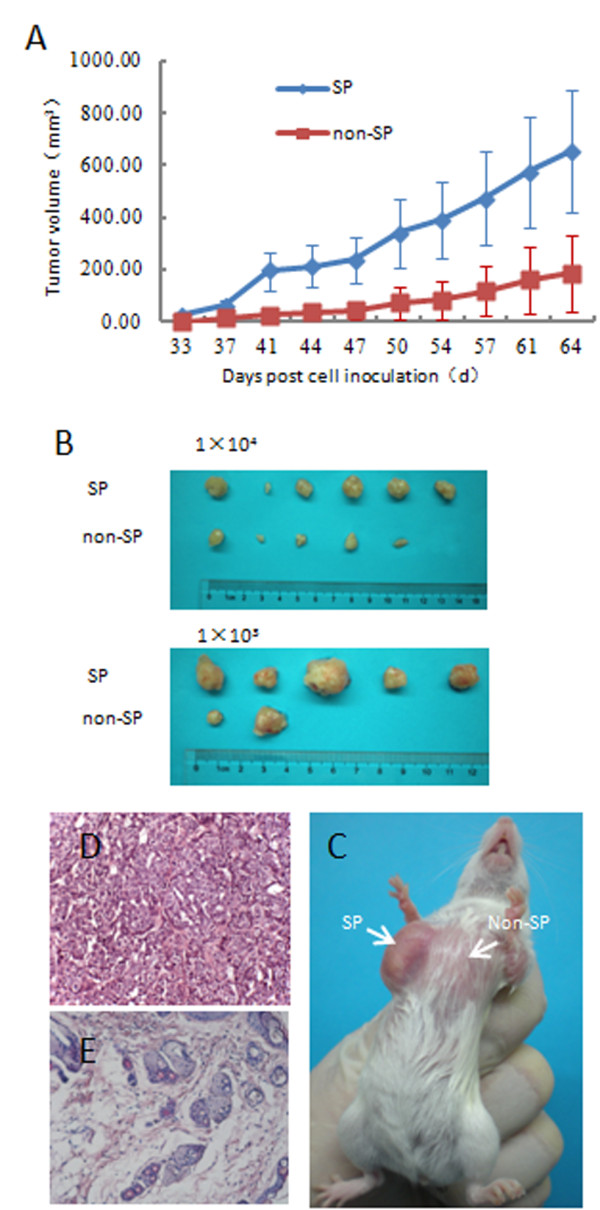
**SP cells were more tumorigenic**. (A) Tumor volumes (mean ± SEM) were plotted for 1 × 10^3 ^cells of each population (SP, non-SP) injected (n = 6 per group). Tumors derived from SP were larger than those from non-SP. (B) Representative tumors due to injection of SP cells (1 × 10^4 ^cells, 1 × 10^3 ^cells) compared with non-SP injection (1 × 10^4 ^cells, 1 × 10^3 ^cells). (C) A representative tumor in a mouse specimen at the SP injection (1 × 10^3 ^cells) site, but not at the non-SP injection (1 × 10^3 ^cells) site. Histology from the SP injection site ((D), Original magnification, ×200) contained malignant cells, whereas the non-SP injection site ((E), Original magnification, ×200) revealed only normal mammary tissue.

SP (P3) and non- SP (P4) cells were isolated by flow cytometry and collected for this experiment (Figure 3A, B). Tumorigenicity assays were performed by injecting MCF-7 unsorted, SP and non-SP cells into NOD/SCID mice. The SP cells showed higher tumorigenicity than the unsorted and non-SP cells (Table 1). Notably, 6 of 6, and 5 of 6 mice inoculated with 10,000, and 1,000 SP cells respectively gave rise to tumors, whereas only 5 of 6, and 2 of 6 inoculations of the same number of the non-SP cells grew tumors, and 5 of 6, and 3 of 6 inoculations of the same number of MCF-7 cells grew tumors. The tumors derived from non-SP cells were smaller than those from SP cells (Figure 4A, B).

**Table 1:** Tumorigenicity of SP Cells in NOD/SCID Xenotransplant Assay

Cells injected/fat pad	Tumors/injections			
	
	5 × 10^6 ^	1 × 10^5 ^	1 × 10^4 ^	1 × 10^3 ^
Unsorted	6/6	5/6	5/6	3/6
SP	---	---	6/6	5/6
Non-SP	---	---	5/6	2/6

Table showing the number of tumors generated in NOD/SCID mouse fat pads by SP, non-SP, and unsorted cells. Tumor formation by 1 × 10^4 ^cells was observed for 6 weeks after injection, whereas tumor formation by 1 × 10^3 ^cells was observed for 9 weeks after injection.

Nine weeks after injection, the injection sites of 1 × 10^3 ^tumorigenic SP cells and 1 × 10^3 ^nontumorigenic non-SP cells were examined by histology. The SP site contained a tumor about 1 cm in diameter, whereas non-SP injection site contained no detectable tumor (Figure 4C). The tumor formed by SP cells showed the typical pathological features of breast cancer (Figure 4D), whereas only normal mouse mammary tissue was observed by histology at the site of non-SP injection (Figure 4E).

### Wnt signaling pathway is activated in tumors derived from SP cells

**Figure 5:**
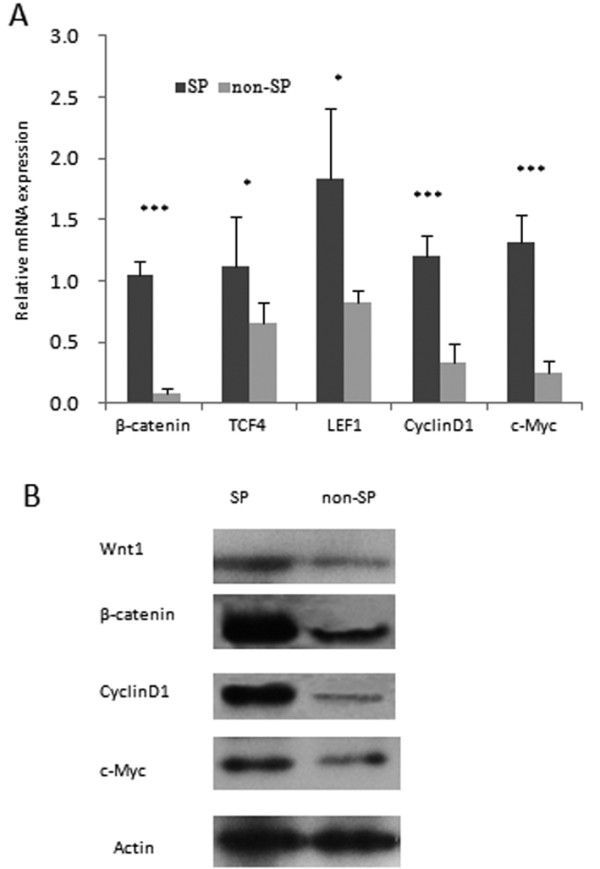
**Wnt/β-catenin was up-regulated in tumors derived from SP cells**.(A) Quantitative RT-PCR analysis revealed that the expression of β-catenin, TCF4, LEF1, CyclinD1 and c-Myc (mean ± SD) were higher in tumors derived from SP than those in tumors from non-SP. These differences were all statistically significant (* *P *< 0.05, ***P < 0.001). (B) Western blotting analysis showed that Wnt1, β-catenin, CyclinD1 and c-Myc in tumors derived from SP expressed higher than those in tumors from non-SP cells. The experiment was run in triplicate.

The key regulator of the Wnt/β-catenin signaling pathway, β-catenin, was first tested. The results showed that the expression of β-catenin was significantly higher in tumors derived from SP cells than that in tumors from non-SP cells at both mRNA and protein level (Figure 5). Wnt1 as an activator of canonical Wnt/β-catenin signaling in MCF-7 cells [32] was tested with other downstream genes and proteins. Quantitative RT-PCR results showed that the main genes of Wnt/β-catenin signaling Wnt1, CyclinD1, c-Myc, TCF4, LEF1 expressed markedly higher in tumors derived from SP compared with those from non-SP (Figure 5A). Moreover, this was associated with a significant increase of the expression of upstream Wnt1, consistent with the up-regulation of lower-stream CyclinD1 and c-Myc at protein level (Figure 5B).

### The effect of CKI on SP cells in vivo

**Figure 6:**
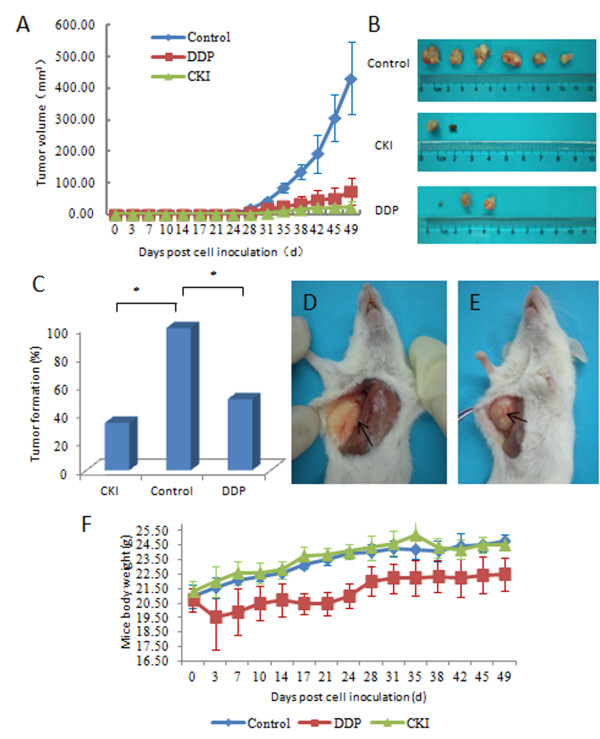
**In vivo efficacy of CKI in the MCF-7 SP xenograft model**. (A) Tumor volumes (Mean ± SEM) were plotted for each group (n = 6 per group). Both CKI and DDP suppressed tumor growth. (B) A representative comparison image of the incised tumors from CKI, DDP, and the control group. (C) The tumor formation rate of the control group was 100% (6/6), while that of CKI group was 33.33% (2/6) and that of the DDP group was 50% (3/6) (* *P *< 0.05). (D) A representative mouse specimen without a tumor from the CKI group. (E) A representative specimen with a tumor from the control group. (F) Schematic outline of mice body weight (mean ± SD). No body weight loss was observed in the CKI group, but a slight body weight loss was observed in the DDP group compared to the control group.

Tumor volumes were measured for up to 7 weeks after inoculation (Figure 6A). Incised tumors among three groups were compared (Figure 6B). Both the CKI and DDP groups showed lower tumor formation rates compared to the control group (*P *< 0.05) (Figure 6C). A representative mouse specimen without a tumor was observed in the CKI group (Figure 6D), whereas a representative specimen with a tumor was observed in the control group (Figure 6E). No body weight loss was observed in the CKI group, whereas a slight body weight loss was observed in the DDP group (Figure 6F).

### Canonical Wnt/β-catenin pathway analysis on CKI and DDP group in vivo

**Figure 7:**
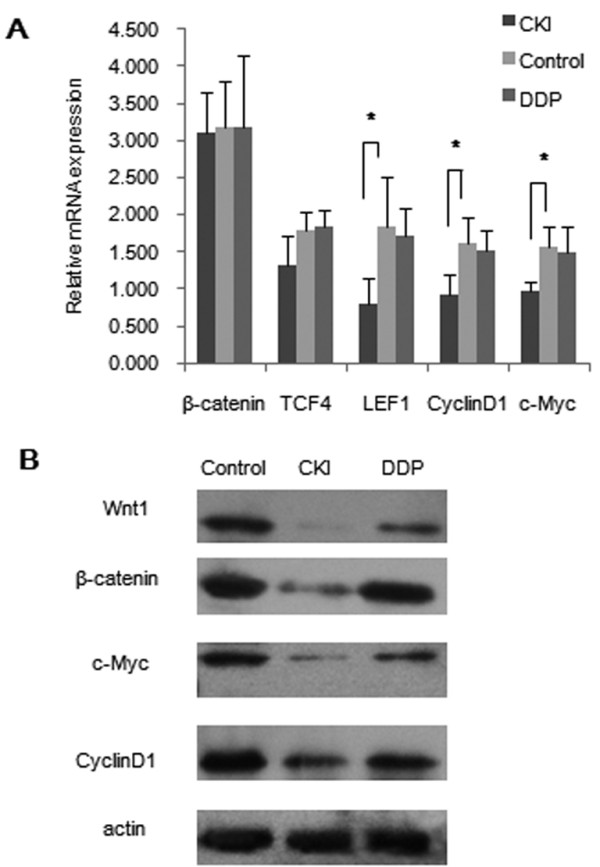
**The Wnt/β-catenin pathway was down-regulated in the CKI group and up-regulated in the DDP group**. a Quantitative RT-PCR analysis revealed that the expression of β-catenin, TCF4, LEF1, CyclinD1 and c-Myc (mean ± SD) were lower in CKI group than those in the control group. Most of the differences were statistically significant (* *P *< 0.05). The expression of β-catenin, TCF4, LEF1, CyclinD1 and c-Myc (mean ± SD) in DDP group were comparable to those in the control group. b Western blot analysis showed that Wnt1, β-catenin, CyclinD1 and c-Myc in the CKI group were significantly lower than those observed in the control group. The protein level of Wnt1, β-catenin, CyclinD1, and c-Myc in DDP group were comparable to those in the control group. The experiment was run in triplicate.

Western blot and RT-PCR analyses were used to investigate whether CKI could down-regulate the expression of the main components of Wnt/β-catenin Pathway. The study found a dramatic decrease of β-catenin with CKI treatment, but the same down-regulation was not observed at the mRNA level. Both the related downstream genes, including TCF4, LEF1, CyclinD1, and c-Myc expressed significantly lower in tumors of CKI group than those of control group as well as the key proteins including wnt1, CyclinD1, c-Myc (Figure 7), which indicated that canonical Wnt/β-catenin signaling pathway was inactive in tumors within the CKI group.

The Wnt/β-catenin Pathway of the DDP group was analyzed at both the protein and mRNA level. The main genes and proteins in DDP group were comparable to those in the control group, suggesting that Wnt/β-catenin Pathway was still active in the DDP group (Figure 7).

## Discussion

How to target CSCs has become a major area of research in recent years. Thus, establishing an appropriate in vivo cancer stem cell model is critical for the study of the treatment of CSCs. Our studies confirmed that SP cells sorted by flow cytometry from human breast cancer cell line MCF-7 showed high expression of CD44^+^CD24^- ^cells and had greater tumorigenicity than non-SP and unsorted cells, which indicates SP cells enrich CSCs. The tumorigenic rate of the mice inoculated with 10,000 SP cells is 100% (6/6), based on which we created a mouse model for the drug intervention study of SP cells. CKI has been widely used in Chinese clinics for many years with the remarkable effects of controlling tumor size and improving the quality of life among cancer patients. But the underlying mechanism has yet to be determined. Our group was the first to show that CKI suppressed cancer-stem like cells (SP) in vitro and in vivo in comparison to the control group.

Wnts are secreted lipid-modified signaling proteins that initiate the canonical Wnt/β-catenin pathway [33], resulting in the accumulation of cytoplasmic (signaling) β-catenin, which are then able to bind the T cell factor/lymphoid enhancer Factor (TCF/LEF) family of transcription factors and to induce the transcriptional activities of targeted genes including CyclinD1, c-Myc, CD44, and matrix metalloproteinase 7 (MMP7), etc [34,35]. In the absence of Wnt signaling, the level of β-catenin is kept low through degradation. The Wnt signaling pathway plays a critical role for the maintenance of CSCs of various cancers [24-26,36-38]. The RT-PCR and western blot analyses showed that Wnt signaling pathway was activated in tumors derived from SP cells, but down-regulated in tumors derived from non-SP cells. It was reported that the aberrant activation of the canonical Wnt/β-catenin signaling pathway is associated with tumor development and progression [23,24,39-41]. Therefore the up-regulation of Wnt signaling pathway correlates with the tumor progression, which explains the high tumorigenicity of SP cells. The results showed that the CKI down-regulated Wnt/β-catenin signaling pathway in vitro and in vivo, but the down-regulation of β-catenin was not observed at the mRNA level in vivo, suggesting that the underlying mechanism is not transcriptional activation but the increased degradation of β-catenin via the destruction complex [42]. Thus, we surmise that the effect of CKI on SP cells may be related to the down-regulation of the Wnt/β-catenin signaling pathway.

The asymmetric division of each CSC allows it to generate one stem cell and another cell that differentiates [43]. So drugs only targeting on differentiated cells will ultimately fail to inhibit tumor growth. Chemotherapeutic drugs are known to be resistant to CSCs which have the capacity to efflux drugs by ABC drug pumps [2,3]. In this study, the DDP suppressed the tumorigenicity of SP cells but the DDP activated the Wnt/β-catenin signaling pathway. Our in vitro study demonstrated that the activation of the Wnt pathway promotes the proliferation and self-renewal of SP cells, and the DDP only inhibits non-SP cells (differentiated cells) leading to the survival of cancer-stem like cells (SP cells) [28], which is also consistent with other studies related to the use of chemotherapeutic drugs [44-46]. Hence, we postulate that the DDP inhibits the differentiated cells derived from SP cells which accounts for 97~98% of MCF-7 cell line leading to a decrease of tumor size, but spares the SP cells endowed with drug-resistance properties and activates the Wnt pathway [44], which requires longer latency period of tumor formation. Further prolonged study is required to demonstrate this.

We also observed that this study has some limitations owing to the use of NOD/SCID mice. In clinical settings, we administered CKI intravenously to cancer patients daily for 2-3 courses (a course consists of 2-3 weeks). Based on this, we injected CKI into NOD/SCID mice i.p. daily. However, the NOD/SCID mice gradually died from a dramatic weight loss about one month post-xenotransplantation in both control group and the CKI group, which didn't occur in the DDP group that was given an injection once a week for three weeks. We attributed this to the severe immune deficiency of NOD/SCID mice which couldn't endure the daily injections of i.p. stimuli. Subsequently, we changed our drug administration to every other day and thereafter mice from CKI group displayed no abnormal weight loss.

## Conclusions

In summary, CKI suppressed MCF-7 SP cells in vitro and in vivo which may be caused by the down-regulation of the Wnt/β-catenin signaling pathway. It suggests that CKI may serve as a novel drug targeting CSCs. In Chinese clinics, we commonly administer CKI to synergizes the therapeutic effects of chemotherapy or radiotherapy. Since CKI specifically suppresses SP cells and cisplatin is known to inhibit non-SP cells, future studies may combine them together to determine the effects on suppressing the tumorigenicity of SP cells. In addition, further studies are warranted to confirm the effects of CKI on cancer stem-like cells of other cancer cell lines and primary carcinomas.

## List of Abbreviations

CSCs: cancer stem cells; SP: side population; CKI: Compound Kushen Injection; NOD/SCID: non-obese diabetic/severe-combined immunodeficient; DDP: cisplatin; HBSS: Hank's balanced salt solution; H&E: hematoxylin and eosin; LEF: lymphoid enhancer factor; TCF: T-cell factor; MMP:matrix metalloproteinase; FACS: fluorescence activating cell sorter; ABC: adenosine triphosphate-binding cassette

## Competing interests

The authors declare that they have no competing interests.

## Authors' contributions

LHS and ZY conceived of the study. XWR did the cell culture, cell isolation, and wrote this paper. XWR, ZZZ and YLL did in vivo experiments. XWR and ZXY did RT-PCR and Western Blot. LHS, ZY, CXY, HBJ, HW, QX and PYX participated in the study design and coordination. All authors read and approved the final manuscript.

## Supplementary Material

Additional file 1 **A representative fingerprint of CKI**. A representative fingerprint of CKI showing 8 common peaks. Peak 3 is Oxymatrine, Peak 4 is Oxysophocarpine, Peak 6 is Matrine, and Peak 7 is Sophocarping. Click here for file
