# Supplementary figures and images for: Compound Kushen Injection suppresses human breast cancer stem-like cells by down-regulating the canonical Wnt/β-catenin pathway
Source: J Exp Clin Cancer Res. 2011 Oct 28;30(1):103. doi: 10.1186/1756-9966-30-103 (PMC3219673; doi:10.1186/1756-9966-30-103)

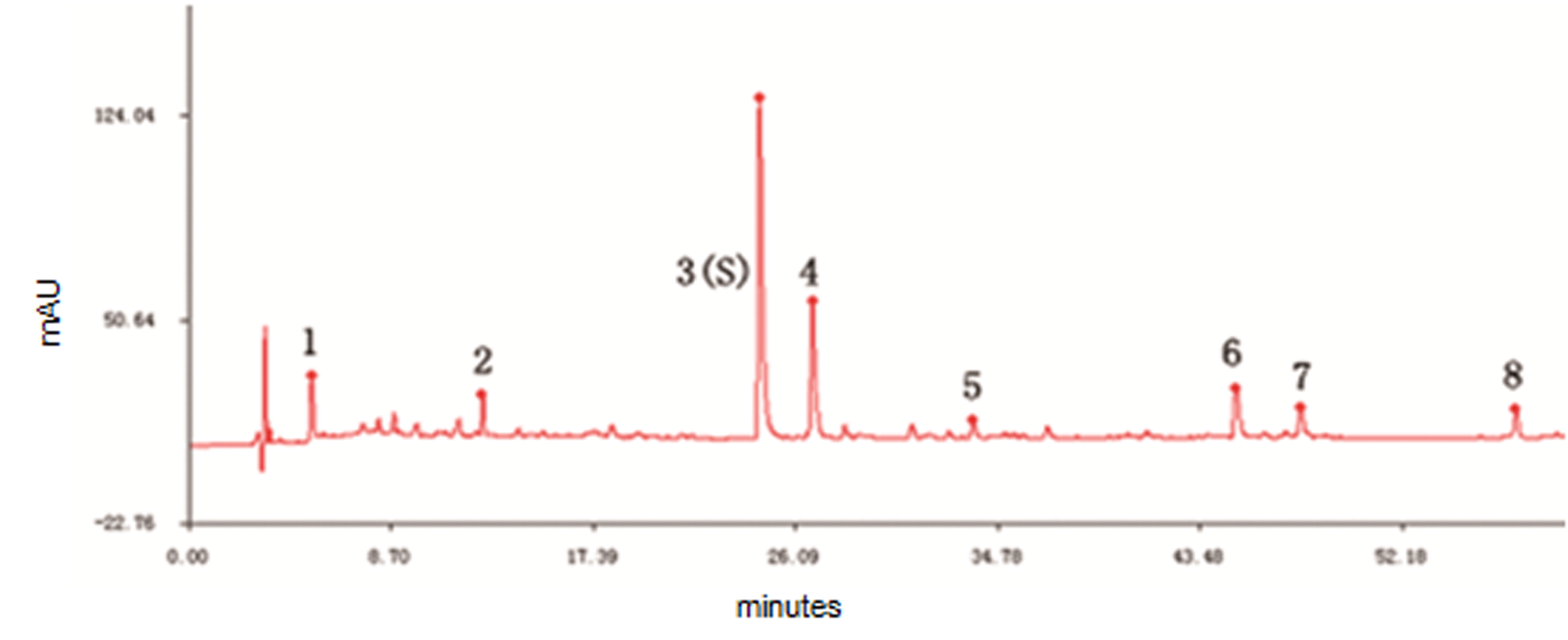

Supplement: Additional file 1 — A representative fingerprint of CKI. A representative fingerprint of CKI showing 8 common peaks. Peak 3 is Oxymatrine, Peak 4 is Oxysophocarpine, Peak 6 is Matrine, and Peak 7 is Sophocarping. [file 1756-9966-30-103-S1.TIFF]
